# The Brocchieri Styptic

**Published:** 1846-03

**Authors:** 


### THE BROCCHIERI STYPTIC.

Our readers have seen notices in the public prints of the Brocchieri water, the styptic powers of which are reported to be equal to any hemorrhage. The carotids of a sheep having been cut, the spouting blood was at once checked by the application of a few drops of the water on a little of the animal's wool. There appears to be something magical in it; but the incredulous profession, as usual, turn a deaf ear to these reports. Dr. Mott says he knew Brocchieri in Paris, and that he was a notorious charlatan. A writer in the Boston Medical Journal compares his "water" to Don Quixote's "balsam of Frierabras," and concludes that the modern remedy was outdone by that of the knight, the virtues of which he thus sets forth to his friend Sancho: "It is a balsam of which I have the receipt by heart, and he that has it need not fear death, nor so much as think of dying by any wound. And therefore, when I shall have made it, and given it to you, all you will have to do is, when you see me in some battle cleft asunder, (as it frequently happens,) to take up, fair and softly, that part of my body which shall fall to the ground, and, with the greatest nicety, before the blood is congealed, place it upon the other half that shall remain in the saddle, taking especial care to make them tally exactly. Then you must immediately give me to drink only two draughts of the balsam, and then you will see me become rounder than any apple."

This wonderful secret was lost, and when men were beginning to felicitate themselves that it had been restored in the "*aqua Brocchieri*," their visions were put to flight by the assurance that the remedy was "*too costly for general use*."

